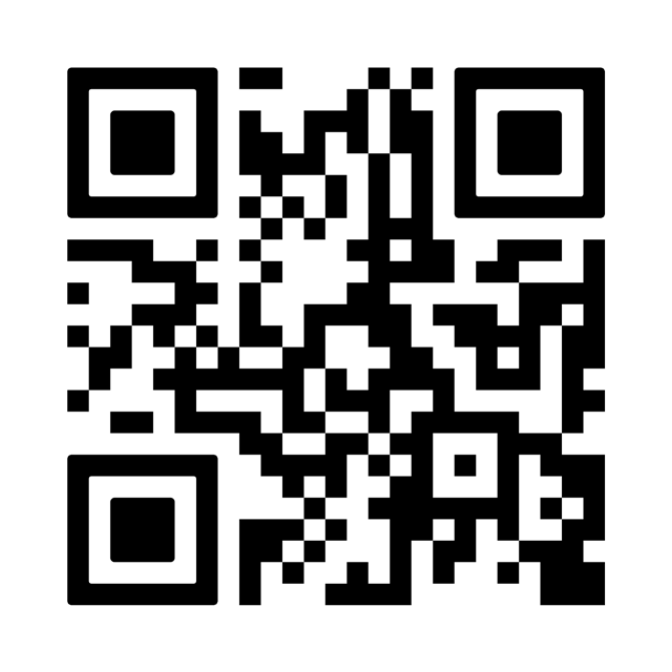# Laparoscopic suture sacrohysteropexy: A meshless uterine-sparing technique for surgical management of uterine prolapse

**DOI:** 10.52054/FVVO.15.2.075

**Published:** 2023-06-30

**Authors:** N Thanatsis, M Ben Zvi, A.S. Kupelian, A Vashisht

**Affiliations:** Urogynaecology and Pelvic Floor Unit, Minimal Access Surgery Team, University College London Hospital, London WC1E 6DB, United Kingdom

**Keywords:** Pelvic organ prolapse, laparoscopic sacrohysteropexy, meshless prolapse surgery

## Abstract

**Background:**

Laparoscopic mesh sacrohysteropexy has been established as an effective, safe, and popular technique to treat uterine prolapse. Nevertheless, recent controversies regarding the role of synthetic mesh in pelvic reconstructive surgery have triggered a trend towards meshless procedures. Other laparoscopic native tissue prolapses techniques such as uterosacral ligament plication and sacral suture hysteropexy have been previously described in literature.

**Objectives:**

To describe a meshless minimally invasive technique with uterine preservation, which incorporates steps from the above-mentioned procedures.

**Materials and Methods:**

We present a case of a 41-year-old patient with stage II apical prolapse and stage III cystocele and rectocele, who was keen to proceed to surgical management preserving her uterus and avoiding the use of a mesh implant. The narrated video demonstrates the surgical steps of our technique of laparoscopic suture sacrohysteropexy.

**Main outcome measures:**

Objective (i.e., anatomic) and subjective (i.e., functional) surgical success on follow-up assessment at least 3 months post-surgery, similarly to every prolapse procedure.

**Results:**

Excellent anatomical result and resolution of prolapse symptoms at follow-up appointments.

**Conclusions:**

Our technique of laparoscopic suture sacrohysteropexy seems a logical progression in prolapse surgery, responding to patients’ wishes for minimally invasive meshless procedures with uterine preservation while at the same time achieving excellent apical support. Its long-term efficacy and safety need to be carefully assessed before it becomes established in clinical practice.

**Learning objective:**

To demonstrate a laparoscopic uterine-sparing technique to treat uterine prolapse without the use of a permanent mesh.

## Introduction

Laparoscopic mesh sacrohysteropexy is one of the NICE (National Institute for Health and Care Excellence) recommended procedures for surgical treatment of uterine prolapse and the most popular uterine-sparing procedure for pelvic organ prolapse (POP) in the United Kingdom (Jha et al., 2018). Studies have shown its excellent anatomic results with a low risk of POP recurrence and re-operation rate (3.7%), as well as its high subjective symptomatic improvement rate (96% of women were satisfied with the procedure) (Kupelian et al., 2016; Izett-Kay et al., 2020). Nevertheless, recent criticism regarding safety of synthetic mid-urethral tapes and transvaginal mesh implants and the associated publicity have created controversies about the role of mesh in contemporary pelvic reconstructive surgery in general. Currently, there is a growing trend towards the use of dissolvable surgical materials.

Laparoscopic suture sacrohysteropexy is an alternative approach suitable for women who are mesh averse and want to preserve their uterus. Other authors have previously described laparoscopic native tissue POP procedures. In 2001, Maher et al. (2001) used non-absorbable sutures to plicate the uterosacral ligaments (laparoscopic uterosacral ligament suspension and culdoplasty). Subsequently, Krause et al. (2006) described the technique of laparoscopic sacral suture hysteropexy. The authors utilised two running non-absorbable sutures to anchor the uterine torus to the right uterosacral ligament and the anterior longitudinal ligament over the sacral promontory. Our technique of laparoscopic suture sacrohysteropexy incorporates surgical steps of both above-mentioned procedures in an effort to achieve optimal and long-lasting apical support. The current video article describes our approach.

## Patients and method

We present a case of a 41- year-old patient with two previous vaginal deliveries, who was referred to our unit due to a sensation of vaginal bulge and pelvic heaviness. She did not report any bothersome lower urinary tract and defecatory symptoms. Her history did not include any medical co-morbidities or any previous abdominal surgeries. Her BMI was normal (24.32 kg/m2). On examination, the patient had a stage II apical prolapse, cystocele and rectocele, with the Pelvic. Organ Prolapse Quantification system (POP-Q) measurements being as follows: Aa +1, Ba +1, C 0, Gh 5, PB 4, TVL 11, Ap +1, Bp +1, D -5. A pelvic ultrasound examination revealed a normal sized uterus with no evidence of any endometrial pathology.

Having already exhausted conservative options (pelvic floor muscle exercises and vaginal support pessaries), the patient decided to proceed to surgical management. She was very reluctant to remove her uterus. In addition, she was keen to avoid vaginal surgery and use of a permanent mesh; therefore, the multidisciplinary team suggested our approach of laparoscopic suture sacrohysteropexy.

A written informed consent for recording and publishing the surgical video was obtained from the patient prior to the procedure.

## Results

The main surgical steps of laparoscopic suture sacrohysteropexy are as follows:

Pelvic survey and ovarian suspension to optimise the view of surgical field.

Identification of ureters and peritoneal relaxing incisions to lateralise the ureters and delineate the uterosacral ligaments (USLs).

Identification of sacral promontory followed by opening of the peritoneum and exposure of the anterior longitudinal ligament (ALL).

Dissection of the peritoneum of the medial pararectal space caudally up to the insertion of the right USL.

In case of a concomitant enterocele, then we proceed to dissection of the rectovaginal space and laparoscopic correction of enterocele using delayed absorbable sutures.

Plication of each USL using delayed absorbable sutures. This step is usually repeated two or three times for each USL.

Midline plication of USLs utilising delayed absorbable sutures (laparoscopic culdoplasty).

Two monofilament non-absorbable sutures are inserted into the uterine torus, then passed into the right USL, anchored to the ALL over the sacral promontory and then passed back to the uterine torus.

All non-absorbable sutures are buried by reperitonealisation.

The procedure, which lasted 115 minutes, was uncomplicated with an estimated blood loss of 100 ml and the patient was discharged on the first post- operative day. Post-operative assessment at 3,12 and 18 months revealed complete resolution of prolapse (POP-Q measurements: Aa -2, Ba 2, C -5, Gh 4, PB 4, TVL 11, Ap -2, Bp -2, D -9) and excellent subjective functional outcomes, as revealed with the use of validated questionnaires (Patient Global Impression of Improvement scale (PGI-I), International Consultation on Incontinence Urinary Incontinence Short Form (ICIQ-UI) and Vaginal Symptoms (ICIQ-VS) questionnaire).

## Discussion

Surgical management of POP has seen a noticeable shift towards minimally invasive abdominal procedures over the last 25 years with the laparoscopic mesh sacrohysteropexy playing a central role in contemporary pelvic reconstructive surgery. Its efficacy and safety as well as the improved outcomes compared to other surgical routes with concomitant hysterectomy have been demonstrated by multiple studies (Meriwether et al., 2018; Izett-Kay et al., 2020); nevertheless, the wide media and political coverage of the safety concerns about synthetic mid-urethral tapes and transvaginal mesh has triggered a trend against synthetic mesh and towards the use of absorbable surgical materials. This is reflected in daily clinical urogynaecological practice: a growing number of patients with POP are keen to undergo a laparoscopic procedure preserving their uterus, but they are reluctant to use a mesh implant.

Our technique of laparoscopic suture sacrohysteropexy was introduced in an effort to respond to patients’ wishes. Its rationale lies in:

(1) Recreation of the crucial Level I support of the apex according to DeLancey. This is achieved through reinforcement of each USL and midline plication of both USLs (culdoplasty).

(2) Providing extra apical support by anchoring the uterine torus-USLs complex to a strong fixation point, i.e., the anterior longitudinal ligament over the sacral promontory.

From our so far experience, the former step suffices in most cases to elevate the apex and correct any POP, whereas the latter step probably ensures the procedure’s durability. The choice of suture material is actually a compromise between the patients’ request to limit or even avoid the use of permanent surgical materials and the need to maximise long-term surgical outcomes. We utilise delayed absorbable sutures for the USL plication and culdoplasty; the efficacy of this step derives mainly from the fibrosis that will develop once you bring the tissues together and not necessarily from the strength or the longevity of the suture itself. Nevertheless, when you suspend the uterine torus to the sacral promontory, you do not expect the fibrosis to play any role, as the tissues approximate only partially, and they do not come in close contact. Therefore, we favour the use of non-absorbable stitches for sacral suspension.

Performing only a laparoscopic USL plication and culdoplasty utilising non-absorbable sutures, Maher et al. (2001) reported 81% subjective success rate (no POP symptoms) and 79% objective success rate (no evidence of POP on examination) at a mean follow-up of 12 months. When Krause et al. (2006) used non-absorbable sutures to anchor the uterine torus to the anterior longitudinal ligament, 94.7% of patients had no objective evidence of POP and 87.8% of them were asymptomatic from POP at a mean follow-up of 20.3 months.

Most women have multi-compartmental rather than isolated apical POP; however, surgical repair of apical descent is often enough to correct certain degrees of cystocele or rectoenterocele. Vaginal examination is routinely performed at the end of a laparoscopic suture sacrohysteropexy and if any residual POP is detected, an anterior or a low posterior vaginal repair and perineorrhaphy will be undertaken. In the current case report, a concomitant anterior or low posterior repair was not deemed necessary, as the patient was found to have minimal only residual cystocele and rectocele.

## Conclusions

Our technique of laparoscopic suture sacrohysteropexy was developed in an effort to offer alternative minimally invasive surgical options to mesh averse women who wish to undergo a uterine sparing prolapse surgery. Undoubtedly, it requires a surgical team competent in both advanced laparoscopic surgery and urogynaecology; however, it is a reasonable evolution of POP surgery responding to patients’ desires for minimally invasive techniques, avoiding a mesh implant and achieving excellent apical support. Long term data especially with regard to its anatomic and functional success but also with regard to its safety are necessary prior to its introduction into daily clinical practice.

## Video scan (read QR)

https://vimeo.com/828975944/1b3c330d56?share=copy

**Figure:**